# Combining Ability for Germination Traits in *Jatropha curcas* L.

**DOI:** 10.1155/2013/935981

**Published:** 2013-10-03

**Authors:** A. K. M. Aminul Islam, Nurina Anuar, Zahira Yaakob, Jaharah A. Ghani, Mohamad Osman

**Affiliations:** ^1^Department of Chemical and Process Engineering, Faculty of Engineering and Built Environment, Universiti Kebangsaan Malaysia, 43600 Bangi, Selangor, Malaysia; ^2^Department of Mechanical and Material Engineering, Faculty of Engineering and Built Environment, Universiti Kebangsaan Malaysia, 43600 Bangi, Selangor, Malaysia; ^3^Faculty of Plantation and Agrotechnology, Universiti Technology Mara (UiTM), 40450 Shah Alam, Selangor, Malaysia

## Abstract

Six parents of *Jatropha curcas* were crossed in half diallel fashion, and the *F*_1_s were evaluated to determine the combining ability for nine germination parameters. The ratio between general combining ability (GCA) and specific combining ability (SCA) variances indicated preponderance of additive gene action for all the characters except germination percentage, time of 50% germination, seedling length, and seedling vigor index. The parents *P*_1_ and *P*_2_ were the best general combiner for most of the characters studied. The cross *P*_1_ × *P*_5_ was the best specific combiner for speed of emergence, germination percentage, germination energy, germination index, and seedling vigor index, the cross *P*_2_ × *P*_5_ for mean germination time, time of 50% germination, and seedling length, and the cross *P*_4_ × *P*_5_ for number of days to first germination. The germination percentage varied from 58.06 to 92.76% among the parents and 53.43 to 98.96% among the hybrids. The highest germination (98.96%) was observed in hybrid *P*_2_ × *P*_4_, and none of the hybrids or parents showed 100% germination. The highest germination index (GI) and seedling vigor index (SVI) were found in hybrid *P*_1_ × *P*_5_ and *P*_2_ × *P*_5_, respectively. The results of this study provide clue for the improvement of *Jatropha* variety through breeding program.

## 1. Introduction

Physic nut (*Jatropha curcas* L., family of Euphorbiaceae) is a common oil seed plant, its seeds contain about 30% of inedible oil, can be found in most tropical and subtropical countries of the world, and can grow up to 5 m height [1]. *Jatropha curcas* is a native of Mexico and Central American region and was later introduced in many parts of the tropics and subtropics of the world [1]. It has low requirements to soil fertility and can grow under low rainfall conditions [2].

*Jatropha curcas* is a multipurpose biodiesel plant adaptable to a wide range of edaphic and climatic conditions [3]. It is easily propagated by both generative (direct seeding) and vegetative (stem cuttings) techniques [1, 3–5], but plants propagated from stem cuttings do not develop a taproot. The plant only develops thin roots unable to grow deep in to the soil, which makes the plants more susceptible to uprooting by strong wind [6]. In agroforestry and intercropping systems, direct seeding is preferable over precultivated *J. curcas* plants, because the taproots of direct seeded plants deeply penetrate the soil layers [7], where it can assess extra nutrient resources and compete less with the roots of other companion crops [1]. Genetic and environmental factors determine germination rate, speed of germination, and vigor of seed and seedling [8]. The germination traits have been found to be complicated, and its inheritance varied among the crops. Speed of germination is genetically correlated to seed dormancy. Genes with additive action conferred high germination speed, while slow germination speed appeared to be induced by genes with pleiotropic effects [9]. Selection for quick germination was unsuccessful, and differences between genotypes were regulated by maternal tissue of seed ball [10]. 

Currently, crop improvement work in this species is limited. Interspecific hybridization has been attempted between different species of *Jatropha* with limited success [11, 12]. Improvement of *Jatropha* can be done through many breeding options like study of combining ability, heterosis breeding, mutation breeding, interspecific hybridization, and genetic transformation [13]. Better understanding and realizing the mode of inheritance of morphoagronomical traits of *J. curcas* in a set of half diallel crosses lead to improve its breeding program [14]. The combining ability and heritability estimation is one of the main strategies for heterosis utilization. Diallel cross analysis leads to a fruitful result for identification of genetic parameters regarding combining ability as well as dominance relationship of the parents. However, very little information is available on combining ability and heritability of germination traits in *J. curcas*. The main objective of the present study was to identify high heterotic parental combination in order to develop hybrid variety with good seed germination quality.

## 2. Materials and Methods

A greenhouse study was conducted at Universiti Kebangsaan Malaysia (UKM) during January to February 2010. Twenty-eight candidate plus plants (CPPs) were selected on the basis of their agromorphological traits from a population of 487 individuals of 20 accessions originated from nine different origins (Swaziland, Cape Verde, India, Thailand, Vietnam, Indonesia, Borneo, Malaysia, and South Africa) and multiplied by stem cuttings to develop materials for genetic diversity study. Parents of the hybridization program were selected through field evaluation followed by genetic diversity study. Six parents (*P*_1_: Indonesia, *P*_2_: Malaysia, *P*_3_: Borneo, *P*_4_: Indonesia, *P*_5_: Cape Verde, and *P*_6_: South Africa) were mated in a half diallel fashion during July to August 2009. The seeds of 6 × 6 half diallel population of *J. curcas* were sown directly in polybags (18 × 10 × 7 cm) containing sand : soil : compost in the proportion of 1 : 1 : 1 at a depth of 3 cm as described by Henning [4]. Watering was done three times per week to continue normal growth of seedlings. The experiments were laid in a completely randomized design with three replications (15 seeds in each). Data on different germination parameters were recorded at 24 hours intervals and continued until no further germination occurred. Seed germination criterion was visible protrusion on the soil surface of at least 0.5 cm of the cotyledon and hypocotyls of the seedlings. The seedlings were evaluated as described in Seedling Evaluation Handbook [15].

Mean germination time (MGT) was calculated based on the equation of Ellis and Roberts [16]. Germination index (GI) was calculated as described in the Association of Official Seed Analysts [15]. Time to 50% germination (*T*_50_) was calculated according to the following formula of Coolbear et al. [17] modified by Farooq et al. [18]:

(1) time  of  50%  germination  =[ti+{(N/2)−ni}(ti−tj)]ni−nj,

where *N* is the final number of germination and *n*_*i*_, *n*_*j*_ are cumulative number of seeds germinated by adjacent counts at times *t*_*i*_ and *t*_*j*_ when *n*_*i*_ < *N*/2 < *n*_*j*_.

Vigor index was calculated according to following formula:

(2) seedling  vigor  index  (SVI) =[seedling  length  (cm)×germination  percentage100].

The speed of emergence was calculated according to following formula:

(3) speed  of  emergence =(number  of  seedlings  emerged  5  days  after  sowingnumber  of  seedlings  emerged  15  days  after  sowing)  ×100.

Germination energy (GE) was determined as the percentage of germinating seeds five days after planting relative to the total number of seeds tested [19].

Number of days taken for first germination was counted from the date of treatment. Numbers of days to first and last germination for each trial were observed. Moreover, measurements of the percentage of germinated seeds were made.

Final germination percentage (GP) and seedling length (SL) were recorded after 15 days of planting as suggested by Dezfuli et al. [20]. For statistical analysis, the data of germination percentage was transformed to arcsin $\sqrt{\left(100/X\right)}$. Experimental data was analyzed by a statistical package SAS, version 9.01 [21]. Treatments means were compared using Tukey's test at 5% level of probability [22]. Combining ability was analyzed by method II (half diallel population with parents) and model I (fixed effect) of Griffing [23].

## 3. Results

The analysis of variance revealed highly significant (*P* < 0.01) differences among the parents and their hybrids for all the germination traits indicating the existence of wider variability among the parental genotypes and the hybrids of *J. curcas*. The GCA and SCA variances were highly significant for all germination traits (Table 1). The GCA variances were higher than SCA variances for five traits, namely, number of days to first germination, speed of emergence, germination energy, germination index, and mean germination time. On the other hand, SCA variances were higher than GCA variances for four traits such as germination percentage, time of 50% germination, seedling length, and seedling vigor index (Table 1).

The highest germination percentage (98.96%) was observed in hybrid combination *P*_2_ × *P*_4_ (Table 2), followed by the hybrids *P*_1_ × *P*_2_ (96.46%). The lowest germination percentage (53.43%) was recorded from the hybrid *P*_5_ × *P*_6_. The highest speed of emergence (70.67) was observed in the parent *P*_1_ and the lowest (4.80) in *P*_6_ (Table 2). Hybrids showed higher speed of emergence as compared to their parents and ranges from 3.73 to 69.47 (Table 2). The highest germination energy (53.44) was observed in the parent *P*_4_ and the lowest (13.27) in *P*_5_. The hybrid combination *P*_1_ × *P*_5_ had the highest (80.00) germination energy followed by the hybrids *P*_1_ × *P*_2_ (76.22). The parents *P*_1_ and *P*_5_ showed the highest (6.00) and lowest (2.78) germination index, respectively, but their hybrids showed an increase in germination index compared to their parents (Table 2). The highest (8.63) germination index was found in hybrid combination *P*_1_ × *P*_5_ and the lowest (2.31) in hybrid *P*_2_ × *P*_4_. The highest mean germination time was observed (10.18 days after first germination) in parent *P*_6_ and the lowest (7.26 days) in *P*_1_ (Table 2). The lowest mean germination time (6.14) was found in the hybrid combination *P*_1_ × *P*_5_ and the highest (12.48) in *P*_2_ × *P*_3_.

The lowest mean germination time was counted in hybrid combinations *P*_1_ × *P*_2_, *P*_1_ × *P*_5_, and *P*_2_ × *P*_5_ (Table 2). The lowest (2.72) and the highest (5.22) *T*_50_ values were observed in the parents *P*_4_ and *P*_6_, respectively. The lowest *T*_50_ value (1.80) was found in the hybrid combination *P*_1_ × *P*_5_ and the highest (5.23) in *P*_4_ × *P*_6_. Most of the hybrids showed lower *T*_50_ value compared to their parents (Table 2). Out of six parents, *P*_1_ produced the tallest seedling compared to other parents (Table 3). The tallest (33.33 cm) seedling was observed in the hybrid combination *P*_2_ × *P*_5_ followed by *P*_6_ × *P*_6_ (Table 2), while the shortest (19.66 cm) seedling in *P*_1_ × *P*_4_. The highest seedling vigor index (24.39) was observed in the parent *P*_4_ and the lowest (14.41) in *P*_5_ (Table 2). Hybrids showed higher seedling vigor index compared to their parents and ranges from 13.30 (*P*_3_ × *P*_6_) to 29.78 (*P*_1_ × *P*_2_) (Table 2).

The parent *P*_1_ was the best general combiner for number of days to first germination (−0.56**), speed of emergence (23.69**), germination energy (14.27**), germination index (1.42**), mean germination time (−1.06**), and time of 50% germination (−0.58**), as it showed the highest significant desirable GCA effects (Table 3). The parent *P*_2_ was the best general combiner for seedling length (1.81**) and seedling vigor index (3.26**) and *P*_4_ for germination percentage (3.68**). The parent *P*_6_ was the poor general combiner for all the germination traits except *P*_4_ for seedling length (Table 3). 

The cross *P*_1_ × *P*_5_ was the best specific combiner for germination percentage (16.10**), speed of emergence (25.17**), germination index (2.77**), and seedling vigor index (5.91**). The best specific combiner for germination energy (30.57**), mean germination time (−2.20**), *T*_50_ (−1.76**), and seedling length (3.36) was *P*_2_ × *P*_5_ (Table 4). The poor specific combiner was the cross combination *P*_4_ × *P*_5_ (Table 4). On the contrary, the poor specific combiners for most of the characters were *P*_1_ × *P*_6_, *P*_2_ × *P*_4_, *P*_3_ × *P*_4_, *P*_4_ × *P*_5_, and *P*_4_ × *P*_6_ (Table 4). The highest negative significant SCA effects (−1.12**) were observed in the cross combination *P*_4_ × *P*_5_ for number of days to first germination and the highest positive significant SCA effects (1.13**) in *P*_4_ × *P*_6_ (Table 4).

## 4. Discussion

Highly significant mean sum of squares due to general and specific combining ability (GCA and SCA) for all the characters indicates that both additive and nonadditive types of gene action were involved for the expression of these characters. The magnitude of SCA variances was higher than that of GCA variances for germination percentage, time of 50% germination, seedling length, and seedling vigor index, which indicates the predominance of the non-additive gene effects for these characters. The remaining characters, namely, number of days to first germination, speed of emergence, germination energy, germination index, and mean germination time, showed higher GCA variances than their respective SCA variances which indicates the predominance of additive gene effect on the expression of these characters. So, it was evident that both additive and dominant genetic components are important for germination parameter in *J. curcas*. Sadeghian and Khodaii [24] also reported additive and nonadditive gene action for germination traits in sugar beet. The GCA effects (*g*_*i*_) represent the additive nature of gene action. Both the nature (direction or sign) and magnitude of *g*_*i*_ are important. Besides, performance of the parent *per se* is also considered together with *g*_*i*_ to select the parent. GCA and SCA variances play significant role in the choice of parents. A parent with higher positive significant GCA effects is considered as a good general combiner.

The parent *P*_1_ was the best general combiner for number of days to first germination, speed of emergence, germination energy, germination index, mean germination time, and time of 50% germination. Again, parent *P*_2_ was the best general combiner for germination percentage, seedling length (cm), and seedling vigor index. The GCA was significantly different among six parents for germination traits in Brassica [25]. Seed vigor traits showed significantly higher GCA value in maize and sorghum [26]. Highly significant GCA effects were also reported for plant height, collar diameter, and number of leaves in nursery stage of *J. curcas* [14]. So, the parent *P*_1_ and *P*_2_ can be selected as a good general combiner and could be used in hybridization program for improvement germination quality of *J. curcas* seed.

The SCA effects denote the highest performance of some specific cross combinations and signify the role of non-additive gene action in the expression of the characters. High SCA effects may arise not only in crosses involving *H* × *H* combinations, but also in those involving *H* × *H* (Plus), *L* (Plus) ×  *H* (plus), and from *L* (plus) ×  *L* (plus). Thus, in practice, some of the low combiners should also be accommodated in hybridization program. Based on SCA effects, it was observed that the cross combination *P*_1_ × *P*_5_ was found as a best specific combination for number of days to first germination, speed of emergence, germination percentage, germination index, and seedling vigor index. The cross combination *P*_2_ × *P*_5_ was the best specific combination for germination energy, mean germination time, time of 50% germination, and seedling length and *P*_2_ × *P*_5_ for number of days to first germination. Smith et al. [27] and Thseng and Hou [28] also observed similar results for genetic control of germination traits. SCA effect is an important criterion for the evaluation of crosses, and highly significant SCA effects were reported for seedling growth in *J. curcas* [14]. The SCA cannot be inherited and is only reflected by the interactions of allele or nonallele from the special parents [29].

## 5. Conclusions

Combining ability studies involving 6 × 6 half diallel populations showed both additive and non-additive gene action in the expression of different characters. The magnitude and direction of the significant GCA effects for the six parents provide meaningful information and clue to the future breeding program. The crosses with desirable specific combining ability could be used for exploitation of heterosis in *J. curcas* for germination and seedling growth. For most of the germination parameters, parents behaved poorer than those of hybrids.

## Figures and Tables

**Table 1:** Combining ability ANOVA for nine germination traits of 6 × 6 diallel populations in *Jatropha curcas*.

Sources	df	NDTFG	GP	SE	GE	GI	MGT	*T*_50_	SL	SVI
GCA	5	1.04**	178.66**	1240.25**	642.48**	5.21**	2.71**	1.44**	9.80**	26.31**
SCA	15	0.75**	238.73**	226.92**	430.92**	2.27**	1.97**	1.48**	10.52**	29.84**
Error	40	0.099	1.88	0.63	0.27	0.02	0.03	0.01	0.70	0.55
GCA : SCA	—	1.39	0.75	5.47	1.49	2.30	1.38	0.97	0.93	0.88

**P < 0.01.

NDFG: number of days to first germination, SE: speed of emergence, GP: germination percentage (%), GE: germination energy, GI: germination index, MGT: mean germination time (days), *T*_50_: time of 50% germination, SL: seedling length (cm), and SVI: seedling vigor index.

**Table 2:** Performance of parents and their hybrids for nine germination parameters in *Jatropha curcas*.

Genotypes	NDTFG	GP	SE	GE	GI	MGT	*T*_50_	SL	SVI/100
Parents									
*P*_1_	4.00	67.23	70.67	50.00	6.00	7.26	5.00	28.50	19.13
*P*_2_	5.33	66.70	6.53	16.22	3.53	9.73	5.00	25.66	17.12
*P*_3_	5.00	92.76	18.63	50.00	5.20	8.85	4.13	25.83	23.97
*P*_4_	4.33	90.33	31.99	53.44	5.76	8.25	2.72	27.00	24.39
*P*_5_	7.67	58.06	5.90	13.27	2.78	9.83	5.08	24.83	14.41
*P*_6_	6.67	70.33	4.80	13.34	2.89	10.18	5.22	24.50	17.23

Hybrids									
*P*_1_ × *P*_2_	4.67	96.46	59.28	76.22	7.63	6.55	2.00	30.83	29.78
*P*_1_ × *P*_3_	5.33	82.10	39.93	33.11	5.61	8.50	2.00	25.83	21.12
*P*_1_ × *P*_4_	4.33	82.60	20.00	60.00	4.66	8.84	4.00	19.66	16.21
*P*_1_ × *P*_5_	4.33	93.10	69.47	80.00	8.63	6.14	1.80	29.33	27.31
*P*_1_ × *P*_6_	4.33	86.96	7.76	43.11	4.37	8.51	4.78	25.66	22.33
*P*_2_ × *P*_3_	5.33	96.40	10.46	56.39	5.21	12.48	4.59	30.33	29.24
*P*_2_ × *P*_4_	5.00	98.96	16.56	16.65	2.31	8.27	4.50	29.16	28.86
*P*_2_ × *P*_5_	5.00	86.90	37.83	73.44	6.35	6.51	2.21	33.33	28.97
*P*_2_ × *P*_6_	5.00	93.00	3.73	36.55	4.36	8.61	5.00	30.83	28.65
*P*_3_ × *P*_4_	5.00	56.10	6.33	13.44	2.63	9.25	5.00	28.83	16.15
*P*_3_ × *P*_5_	5.00	80.33	8.26	53.31	4.19	8.00	4.73	28.33	22.76
*P*_3_ × *P*_6_	5.33	56.23	6.00	23.47	2.59	9.56	5.00	25.66	13.30
*P*_4_ × *P*_5_	4.00	90.00	3.80	30.00	3.83	10.49	5.00	23.66	23.10
*P*_4_ × *P*_6_	6.33	80.66	4.23	16.39	3.73	9.15	5.23	24.66	19.90
*P*_5_ × *P*_6_	4.33	53.43	12.50	26.55	2.46	9.75	4.64	31.66	16.94
MSD (0.05)	1.69	7.39	4.29	2.81	0.80	0.91	0.46	4.52	3.99

MSD: minimum significant difference, NDFG: number of days to first germination, SE: speed of emergence, GP: germination percentage (%), GE: germination energy, GI: germination index, MGT: mean germination time (days), *T*_50_: time of 50% germination, SL: seedling length (cm), and SVI: seedling vigor index.

**Table 3:** GCA effects for nine germination traits of 6 × 6 diallel populations in *Jatropha curcas*.

GCA effects	NDTFG	GP	SE	GE	GI	MGT	*T*_50_	SL	SVI/100
*P*_1_	−0.56*	2.02**	23.69**	14.27**	1.42**	−1.06**	−0.58**	−0.38	0.17
*P*_2_	0.03	5.70**	−0.91*	1.67**	0.17*	0.04	−0.12**	1.81**	3.26**
*P*_3_	0.07	−0.36	−4.99**	0.18	−0.12	0.49**	0.05	−0.34	−0.39
*P*_4_	−0.26	3.68**	−4.17**	−4.37**	−0.36**	0.12	−0.01	−1.17**	−0.08
*P*_5_	0.32	−4.96**	−0.57	1.44**	−0.07	−0.13	−0.08*	0.83*	−0.72*
*P*_6_	0.40*	−6.08**	−13.05**	−13.19**	−1.04**	0.54**	0.74**	−0.74*	−2.26**
SEd (gi-gj)	0.16	0.68	0.40	0.26	0.07	0.08	0.04	0.42	0.37

*P < 0.05; **P < 0.01.

NDFG: number of days to first germination, SE: speed of emergence, GP: germination percentage (%), GE: germination energy, GI: germination index, MGT: mean germination time (days), *T*_50_: time of 50% germination, SL: seedling length (cm), and SVI: seedling vigor index.

**Table 4:** SCA effects for nine germination traits of 6 × 6 diallel populations in *Jatropha curcas*.

SCA effects	NDTFG	GP	SE	GE	GI	MGT	*T*_50_	SL	SVI
*P*_1_ × *P*_2_	0.13	8.82**	15.32**	20.52**	1.53**	−1.22**	−1.48**	2.07*	4.40**
*P*_1_ × *P*_3_	0.76*	0.50	0.06	−21.09**	−0.2	0.28	−1.64**	−0.79	−0.61
*P*_1_ × *P*_4_	0.09	−3.03*	−20.70**	10.34**	−0.90**	0.99**	0.41**	−6.12**	−5.83**
*P*_1_ × *P*_5_	−0.49	16.10**	25.17**	24.53**	2.77**	−1.46**	−1.71**	1.55*	5.91**
*P*_1_ × *P*_6_	−0.58*	11.08**	−24.05**	2.28**	−0.52**	0.23	0.46**	−0.56	2.46**
*P*_2_ × *P*_3_	0.17	11.12**	−4.80**	14.78**	0.65**	3.16**	0.48**	1.53*	4.42**
*P*_2_ × *P*_4_	0.17	9.65**	0.47	−20.41**	−2.01**	−0.68**	0.45**	1.19	3.73**
*P*_2_ × *P*_5_	−0.41	6.22**	18.14**	30.57**	1.74**	−2.20**	−1.76**	3.36**	4.47**
*P*_2_ × *P*_6_	−0.49	13.44**	−3.48**	8.31**	0.72**	−0.77**	0.20*	2.42*	5.70**
*P*_3_ × *P*_4_	0.13	−27.16**	−5.68**	−22.12**	−1.40**	−0.16	0.78**	3.01**	−5.33**
*P*_3_ × *P*_5_	−0.45	5.72**	−7.34**	11.93**	−0.13	−1.16**	0.60**	0.51	1.92*
*P*_3_ × *P*_6_	−0.20	−17.27**	2.87**	−3.27**	−0.77**	−0.27	0.04	−2.60**	−6.00**
*P*_4_ × *P*_5_	−1.12**	11.34**	−12.64**	−6.84**	−0.25	1.71**	0.91**	−1.33	1.94**
*P*_4_ × *P*_6_	1.13**	3.12*	0.27	−5.81*	0.61**	−0.31*	0.33*	−0.76	0.29
*P*_5_ × *P*_6_	−1.45	−15.470	4.94	−1.46	−0.94	0.53	−0.18	4.24	−2.04
SEd (Sij-Sik)	0.42	1.81	1.05	0.69	0.20	0.22	0.11	1.11	0.98
SEd (Sij-Skl)	0.39	1.68	0.97	0.64	0.18	0.21	0.1	1.03	0.91

*P < 0.05; **P < 0.01.

NDFG: number of days to first germination, SE: speed of emergence, GP: germination percentage (%), GE: germination energy, GI: germination index, MGT: mean germination time (days), *T*_50_: time of 50% germination, SL: seedling length (cm), and SVI: seedling vigor index.
